# Evaluation of the efficacy and safety of the use of acupuncture for the adjuvant treatment of patients with post-stroke cognitive impairment: protocol for a randomized controlled trial

**DOI:** 10.1186/s13063-020-04656-w

**Published:** 2020-08-28

**Authors:** Ruijia Liu, Xudong Yu, Jisheng Wang, Ye Liu, Bowen Liu, Xinwei Li, Yue Wang, Tianyu Cao, Hongwei Yuan

**Affiliations:** 1 Graduate School of Beijing University of Chinese Medicine, Beijing, China; 2 The First Department of Neurology, Dongzhimen Hospital, Beijing University of Chinese Medicine, Beijng, 100700 China; 3 Dongzhimen Hospital, Beijing University of Chinese Medicine, Beijng, 100700 China; 4 The Department of Acupuncture, Dongzhimen Hospital, Beijing University of Chinese Medicine, Beijng, 100700 China

## Abstract

**Background:**

Post-stroke cognitive impairment (PSCI) is a series of syndromes that meet the diagnostic criteria for cognitive impairment that appear after a stroke. The treatment of PSCI with oral drugs alone is not ideal and has obvious side effects. Therefore, complementary and alternative treatments are needed for patients with insufficient or significant side effects of oral medications. Therefore, we will evaluate the clinical effectiveness and safety of acupuncture in the treatment of PSCI.

**Methods/design:**

In this study, patients will be randomly divided into two groups. Intervention group: acupuncture combined with oral medication. Control group: Western medicine treatment plan. All participants will continue to receive conventional drug treatment. The selection of outcomes will be evaluated by Mini Mental State Examination Scale (MMSE) at week 12. The scale will be conducted by two well-trained reviewers who will conduct joint MMSE inspections on participants. The test time will be selected 3 days before treatment and once 4 weeks after treatment. After the MMSE test, the two raters scored independently, and the average of the two was used as the final score.

**Discussion:**

This trial may provide evidence regarding the clinical effectiveness, safety, and cost-effectiveness of acupuncture for patients with PSCI.

**Trial registration:**

ClinicalTrials.gov ChiCTR2000029926. Registered on 17 February 2020 http://www.chictr.org.cn/showproj.aspx?proj=49356

## Background

Cognitive function is composed of multiple cognitive areas such as memory, computing power, time orientation, space orientation, structural ability, execution ability, language understanding ability, and expression application ability [1]. Cognitive impairment refers to a series of syndromes with varying degrees of cognitive impairment from mild cognitive impairment to dementia caused by various reasons. These behavioral and emotional disorders have become important diseases affecting the health and quality of life of middle-aged and elderly people, as well as important causes of disability for patients [2]. The disease places a heavy burden of care and economic burden on society and families. Stroke can cause multiple types and varying degrees of cognitive dysfunction. The definition of post-stroke cognitive impairment (PSCI) was also clarified in “the 2016 Chinese Expert Consensus on Post-Stroke Cognitive Impairment Management”. It points out that PSCI is a series of syndromes that meet the diagnostic criteria for cognitive impairment that appear after a stroke [3, 4]. It highlights the potential causal relationship between stroke and cognitive impairment and the relevance of clinical management between the two. PSCI is a common and serious complication after stroke, with a high incidence, which seriously affects the quality of life and prognosis of patients. In addition, the survival time of patients is significantly reduced, and it can increase the risk of recurrent stroke, thereby placing a heavy burden on society and families [5]. The current treatments for PSCI are oral acetylcholinesterase inhibitors, anti-glutamate drugs, antioxidants, non-steroidal anti-inflammatory agents, and hormone replacement therapies. Recent studies have shown that the anticholinergic function of such drugs has certain limitations and can have a certain effect on patients’ memory [6, 7]. The treatment of PSCI with oral drugs alone is not ideal and has obvious side effects.

**Fig. 1:**
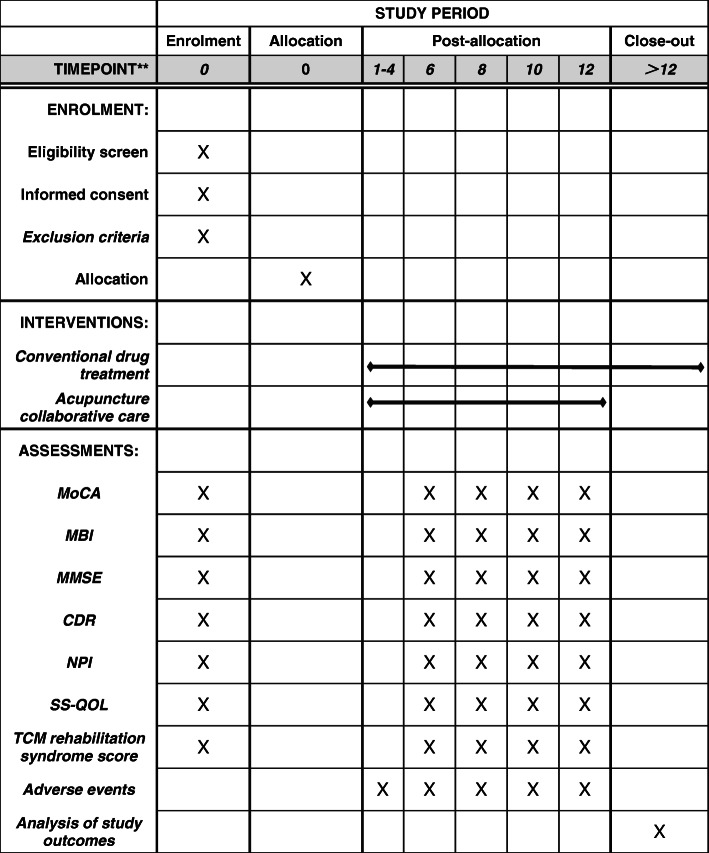
SPIRIT figure for the schedule of enrollment, interventions, and assessments. Abbreviations*: MOCA* Montreal Cognitive Assessment Scale, *MBI* Modified Barthel Index Rating Scale, *MMSE* Mini Mental State Examination Scale, *CDR* Clinical Dementia Rating Scale, *NPI* Neuropsychiatric Inventory, *SS-QOL* Stroke-specific quality of life scale, *SPIRIT* Standard Protocol Items: Recommendations for Interventional Trials, *TCM* traditional Chinese medicine

Acupuncture, as an important part of the appropriate technology of traditional Chinese medicine (TCM), is currently the most commonly used complementary and alternative treatment [8].. Acupuncture has a significant regulating effect on human psychology. Modern studies have found that acupuncture can improve cognitive function by affecting cerebral blood flow and hemorheology, protecting the internal structure of brain nerve cells [9]. The theory of TCM holds that acupuncture can regulate the balance of Qi and blood in the human body, it can improve human function. Acupuncture is becoming more and more popular with clinicians and patients for its unique advantages of simplicity, low cost, and significant efficacy. Based on the current needs of patients with PSCI who have poor response to conventional treatment and obvious adverse reactions, as well as the need for clinical research on the level of evidence for this effect, it is worthwhile to further explore the treatment model of acupuncture for PSCI. Therefore, we will evaluate the clinical effectiveness and safety of acupuncture in the treatment of PSCI. In this manuscript, we describe the rationale and the detailed methodology of the trial. This protocol is guided by the Standard Protocol Items: Recommendations for Interventional Trials (SPIRIT) (Fig. 1 and Additional file 1: Table S1).

## Methods/design

### Study design and setting

In this study, the tested patients will be randomly divided into two groups: intervention group—acupuncture combined with oral medication and control group—conventional western medicine treatment. Patients who meet the conditions of inclusion and exclusion first complete informed consent, will be randomly grouped, and be stratified according to the clinical treatment period. For specific stratification factors and reasons, please refer to the statistical analysis plan “Sample Size Estimation”. The intervention time will be 12 weeks. A 7-day treatment observation and a 28-day follow-up observation will be carried out, and the evaluator evaluated blindly. During the observation period, if a patient meets the criteria for release of isolation and discharge, or dies or voluntarily withdraws, the observation of the patient shall be suspended; after the observation period, patients who do not meet the criteria for release of isolation and discharge will be still hospitalized for standard treatment. In addition, we will implement a clinical surveillance system to ensure research quality. The trial work plan is summarized in Fig. 1.

### Inclusion/exclusion criteria

We aim to recruit patients with PSCI who feel down in the dumps despite standard medical therapy. Patient inclusion criteria: (1) a clear history of ischemic stroke or hemorrhagic stroke was confirmed by CT/MRI examination of the head and (2) memory and/or cognitive and executive function impairments. Cognitive dysfunction will have been determined after Mini Mental State Evaluation (MMSE) score. If the MMSE score is less than 27 points, it can be considered as cognitive dysfunction; (3) age will be limited to between 40 and 75 years of age, regardless of gender; (4) stable condition, clear awareness, no aphasia, can take drugs orally; (5) no severe comorbidities, no major depression; and (6) agree and sign the patient’s informed consent.

Exclusion criteria: (1) computer computed tomography (CT) or magnetic resonance imaging (MRI) shows that in addition to the responsible lesion, there are other lesions unrelated to the lesion or severe brain atrophy or white matter porosity; (2) people with a previous history of mental retardation; (3) patients with previous mental illness and/or with epilepsy; (4) unstable vital signs, diabetes, blood patients with disease or tumor; (5) patients who will have been taking sedatives for a long time; (6) people with a history of allergies to drugs and food; and (7) people who will have been identified with a cognitive impairment before a stroke.

### Randomization and blinding

The attending physician will determine eligible patients based on the inclusion and exclusion criteria. The attending physician will obtain informed consent and refer participants to the study coordinator, who will randomly assign them to the intervention or control department. The research coordinator will keep a random list generated in advance by a biostatistician using a computerized random number generator and will assign treatment plans accordingly. The attending physician who will recruit participants will not understand the order of allocation. The biostatistician and research coordinator will keep the random list until the end of the research to ensure that the assignment is hidden. Therefore, the data analyst will be unaware of the allocation. Participants will be instructed not to disclose the allocation to the attending physician.

### Control group

#### Basic treatment of stroke

Refer to “Guidelines for the Prevention and Treatment of Cerebrovascular Diseases in China” (Department of Disease Control, Ministry of Health, Neurological Society of Chinese Medical Association, 2005. Control blood pressure, use individualized treatment to stabilize blood pressure below 135/85 mmHg or within normal range, select appropriate blood glucose hypoglycemic drugs, control blood sugar within the normal range, select lipid-lowering drugs according to triglyceride and cholesterol, antiplatelet aggregation, symptomatic treatment, prevention, and treatment of complications). A question that needs to be highlighted: regarding the application of antiplatelet drugs, we will apply them according to clinical practice. For patients with ischemic stroke, we will give antiplatelet aggregation drugs. For patients with hemorrhagic stroke, we will not give antiplatelet drugs.

Patients will be given corresponding symptomatic drugs (control of blood pressure, blood sugar, etc.) and regular rehabilitation training (sitting balance training, standing balance training, transfer training, walking training, occupational therapy, etc.). According to the different results of each patient’s cognitive function assessment, take corresponding, one-to-one cognitive function training, including (1) memory training back number, back number, short text retelling, word pairing, etc., and guide patients to use association memory techniques such as methods, segmentation methods and story-writing methods to improve memory effects and guide patients to use their own auxiliary equipment, such as notebooks, cards, and electronic notebooks to assist in the completion of daily affairs; (2) attention training visual tracking, guessing games, computer games, etc.; (3) computational training: designing some content related to daily life for patients to perform calculations, such as simulating shopping and grocery shopping in supermarkets; (4) arrangement and daily routines for execution and problem-solving life-related problems for patients to solve, such as cakes and dressing; (5) apraxia, agnosia training, various sensory stimulation of the affected limb, standing on the affected side, speaking to the patient, etc. The patient repeatedly trains to identify items, shapes, colors, etc. and makes full use of other sensory stimuli such as tactile sensation and hearing. For patients with apraxia, the various movements of daily life are decomposed and practiced, and then continuous movements are trained, and the patients are guided by hand to carry out activities. For patients with mental and motor apraxia, use verbal guidance and use their subconscious movements. Cognitive considerations: (1) avoid analgesics as much as possible during the training of cognitive impairment; (2) create a stimulating atmosphere as much as possible, often give the patient a certain stimulus, let the patient touch the environment, and even consider the position of the bed, should be placed in a prominent position, the patient should not be isolated in the ward; and (3) the patient’s psychological barriers should be lifted. In addition to cognitive problems, patients with cognitive impairment may also have different psychological conditions, such as depression and depression. Only by lifting these psychological barriers can the interest of the patients be stimulated and the confidence of the patients can be improved.

### Intervention group

In this group of patients, we will give the same treatment as the control group. At the same time, on this basis we will give acupuncture treatment. (1) Head acupuncture treatment. Acupoint selection: *Baihui, Sishencong, Shenting,* and *Benshen*, with mental behavior symptoms, plus *Neiguan and Shenmen*. Refer to the sixth edition of acupuncture for acupoint positioning. Operation method: generally take a sitting position, depending on the condition, you can also use the supine or lateral position, using Huatuo brand 0.30 mm diameter, 25-mm-long needle (Suzhou Medical Supplies Factory Co., Ltd.). The needle is about 30° from the scalp, twist the needle diagonally along the selected acupuncture point, the depth of the acupuncture is suitable to reach under the cap-shaped aponeurosis, and twist continuously for 2–3 min quickly, and then connect GM-101 type acupuncture instrument, continuous wave, tolerated by the patient as a degree, changed to sparse and dense wave after 15 min. Remove electroacupuncture after 30 min and continue to retain the needle for 1 h; during needle retention, twist the needle every 30 min. Once a day, five times a week for 4 weeks. (2) Moxibustion treatment. Acupoint selection: *Shenting, Baihui, Shendao, Fengfu, Xinyu,* and *Ganshu*. Operation: use mild moxibustion. First, assist the patient to take the prone position, fully expose the moxibustion site, and then place the moxa stick into the moxibustion device head, aim at the acupoints of Shenting, Baihui, Shinto, Fengfu, Xinyu, and Ganshu, wait for the local skin to be red, have a warm and comfortable feeling, last for 30 min. Adjust the height of the moxibustion head in time according to the patient’s feelings, observe the patient’s local skin color, and avoid blisters caused by excessive temperature, five times/week for 4 weeks.

We will use sterile disposable stainless steel needles of 0.25 mm diameter, 25 mm or 40 mm long and 0.30 mm diameter, 50 mm or 70 mm long, depending on the acupuncture points. In addition to the main acupuncture points specific for the treatment of PS CI, the TCM physicians will be allowed to make minor adjustments to the acupuncture points in view of the differing constitution of the patients as per the holistic treatment philosophy of TCM. Therefore, the number of needles to be inserted per subject per session will differ. The needles will be inserted from 0.25 to 1 in. deep depending on the acupuncture points. After eliciting the de qi response, the needles will be left in place for 30 min. The needles will be stimulated manually every 10 min. The acupuncture treatment will consist of a total of 10 sessions (or 2 courses) in total. Each course of treatment will consist of 5 acupuncture sessions held over 2 weeks, each session lasting 30 min. The patient will have a break of at least 3 days and up to 1 week in between each course of acupuncture. TCM physicians will document components of treatment and adherence in standardized logbooks.

The anatomical location of each acupuncture point is as follows: Baihui is located in the middle of the top of the head, in the cap-shaped aponeurosis; around it, there are left and right superficial temporal arteries and veins anastomotic network, as well as the branches of the greater occipital nerve and the frontal nerve. Sishenchong is also located in the galea aponeurosis; it is surrounded by occipital arteries, veins, superficial temporal arteries, venous top branches and supraorbital arterial and venous anastomotic nets, great occipital nerve, auricular cervical nerve, and supraorbital nerve branches. Shenting is located at the junction of the left and right frontal muscles; it is surrounded by frontal arteries and veins, as well as frontal nerve branches. Benshen is in the frontal muscle; it is surrounded by supraorbital arteries, veins, supraorbital nerves, and superficial temporal arteries and veins. Neiguan is located between the palmar longus tendon and the flexor carpi radialis tendon. Its deep part is the pronator; it is surrounded by the median forearm vein, the median artery, the anterior arteries and veins between the bones, and the inner and outer cutaneous nerves of the forearm. There are median nerve trunk and anterior interosseous nerve distribution. Shenmen is located between the flexor carpi ulnaris and the superficial digita flexor muscles, and the deep layer is the deep digita flexor; underneath it is the ulnar artery passing through; it is surrounded by the medial forearm cutaneous nerve.

### Primary outcomes

The selection of outcomes will be evaluated by Mini Mental State Examination Scale (MMSE) [10]. This scale will be conducted by two trained assessors who performed a combined MMSE examination of the patient, using conversation and observation. After the MMSE test would have been over, the two raters independently will score and take the average of the two as the final score. According to the international agreement MMSE score, the scale will include the following 7 aspects: time-oriented force, place-oriented force, immediate memory, attention and computing power, delayed memory, language, and spatial perception. A total of 30 items, each answer is worth 1 point, the answer is wrong or do not know the answer 0 points, the total scale of the scale is 0–30 points. The test results will be closely related to cultural level. Scoring reference: scores 27–30 are normal, scores < 27 can be considered cognitive impairment, scores 21–26 are mild cognitive dysfunction, scores 10–20 are moderate cognitive dysfunction, and a score of 0–9 is considered severe cognitive impairment. The primary and secondary outcome measures are shown in Table 1.

**Table 1:** Treatment schedule and outcome measures

Items	Before treatment	Treatment period, twice a week, 8 times in total (treatment starts within 1 week after registration)				Post observation period
Time point	Registration	Week 1–4	Week 6	Week 8	Week 12	2 weeks later treatment completion
Inclusion criteria	√					
Exclusion criteria	√					
Informed consent	√					
Confirmation of subjective symptoms	√	√	√	√	√	√
Mini Mental State Examination Scale	√		√		√	√
Traditional Chinese Medicine Syndrome Score	√		√		√	√
Observation of adverse events		√	√	√	√	√

### Secondary outcomes

For the secondary outcomes, we will use the Montreal Cognitive Assessment Scale (MOCA) [11]. It is an assessment tool for rapid screening of cognitive abnormalities. It includes 11 inspection items in 8 cognitive areas including attention and concentration, executive function, memory, language, visual structure skills, abstract thinking, computing, and orientation. The total score is 30 points and ≥ 26 points are normal. It has high sensitivity, covers important cognitive areas, and has a short test time, which is suitable for clinical application. However, it is also affected by education level, differences in cultural background, the examiner’s skills and experience in using MoCA, the environment of the examination, and the emotional and mental state of the testee will all affect the score, and for mild cognitive function, obstacles are more sensitive, as education level will be considered to be the most independent factor affecting the MOCA score. In order to correct the bias caused by education level, the total score is increased by 1 point for subjects with less than 12 years of education. For subjects with 4–9 years of education, the total score is increased by 2 points, and for those with 10–12 years of education, the score is increased by 1 point. This can better correct the bias caused by low education levels.

### Instruments and definitions

The MMSE compiled by Foistein and others in 1975 is one of the most influential and widely used screening scales at home and abroad. The scientific and rationality of this scale in predicting the progress of cognitive function in stroke patients has been fully confirmed. MMSE is a screening scale that mainly evaluates cognitive areas such as orientation, instantaneous memory, calculated attention, delayed recall, ability to understand and use, and language function. This scale is generally used for preliminary screening tests for cognitive function. In clinical practice, the MMSE scale is simple, time-saving, and easy to operate. The scale has high sensitivity and specificity for detecting dementia and has good validity and reliability. However, it also has the disadvantages of rough scores and incomplete evaluation. Because the education level of patients will affect their assessment results, false-positive results may be clinically obtained. Other studies have shown that the MMSE scale scoring method is too simple and leads to poor sensitivity. The sensitivity for screening for mild cognitive impairment (MCI) is only 1–9%, and there is a “ceiling effect”. Therefore, other scales need to be further improved and supplemented.

The Montreal Cognitive Assessment Scale (MoCA) was developed by Canadian Nasreddine and others based on clinical experience and with reference to MMSE (Concise Mental State Examination) cognitive items and scores. The Montreal Cognitive Assessment Scale adds assessments of functions such as executive function, visual spatial function, fluency of words, digital forward and backward. Where MMSE cannot detect cognitive impairment, MOCA can be an effective and simple screening tool. In addition, the patient’s early MOCA score can predict the risk of cognitive impairment and risk in the future and has certain predictive validity. It has certain guiding value for clinical judgment of patients’ cognitive prognosis.

### Safety

Adverse reaction (AE) refers to the occurrence of harmful reactions unrelated to the purpose of treatment during the process of preventing, diagnosing, or treating diseases by applying drugs according to normal usage and dosage. Its specific occurrence condition is to use the medicine in the normal dosage and normal usage and exclude the reaction caused by the drug abuse, excessive misuse, use of the medicine in accordance with the prescribed method, and quality problems. Efficacy indicators reflect the effectiveness of interventions, while adverse events reflect intervention safety. In this study, adverse events refer to adverse medical events that occur after a patient or subject in a clinical trial receive an intervention, but are not necessarily causally related to the intervention. Adverse events and adverse reactions: the concept is different. Adverse reactions are directly related to the intervention, and the scope of adverse events covers adverse reactions.

### Sample size justification

According to clinical experience, the effective rate of PSCI in the intervention group was P1 = 0.80. The PSCI effective rate of the control group was P2 = 0.65 [(two-sided type I error rate of 0.05, *α* = 0.05); (one-sided type I error rate of 0.10, *β* = 0.10)]. Substituting *f* (*α*, *β*) into the formula is as follows: *n*1 = *n*2 = 10.5 × (0.8 × 0.2 + 0.65 × 0.35) ÷ 0.152 = 75.The lost follow-up rate of patients will be controlled at 10%. *n*1 = *n*2 = 83, that is, 83 cases will be taken from each of the intervention group and the control group. According to Cohen, this effect size is considered “moderate”.

### Data collection and management

The case report form (CRF) is a paper version, and the CRF is a carbonless copy, in duplicate, to be completed by the investigator. The inspector (CRA) shall conduct an on-site inspection of the CRF, and the problems discovered shall be verified and signed by the researcher after verification. After the completed paper version of the CRF is reviewed and signed by the CRA and the main investigator, the first copy is submitted to the data management unit. After the CRF is submitted, a logical data review is performed by the data management team. If there is any doubt about the data, the data administrator will send an “electronic data inquiry” to the CRA and the researcher and send the inquiry to the researcher through the CRA. The researcher should answer and return as soon as possible. All error content and modification results shall be recorded and kept properly. Paper CRFs should be properly recorded and kept between researchers, CRAs, and data stewards.

### Statistical analysis

Statistical analysis will be performed using SPSS 25.0 software for statistical analysis. The normality of the measurement data is tested. The data obeying the normal distribution is Student’s *t* test, which is expressed by mean ± standard deviation. The data not obeying the normal distribution is rank sum test and marginal homogeneity test. Count data are expressed by rate and composition ratio, and comparison is performed by chi-square test. Repeated measurement data are expressed by mean ± standard deviation, intra-group comparison is performed by analysis of variance of repeated measurement data, and inter-group comparison is by multivariate analysis of variance (MANOVA). *P* ≤ 0.05 indicates that the difference is statistically significant.

### Ethics and dissemination

This study has been approved by the Ethics Committee of Dongzhimen Hospital Affiliated to Beijing University of Chinese Medicine (reference number DZMEC-KY-2019-26). Independent clinicians and biostatisticians with extensive research experience in clinical trials will serve as the Data and Safety Monitoring Committee. The Ethics Committee of Dongzhimen Hospital Office may perform random audits to ensure that relevant regulations and guidelines are met. Study participation is voluntary and can be discontinued at any time, and deciding not to take part will not affect a patient’s care. Protocol amendments, adverse effects reporting, and annual review will be overseen by the Ethics Committee of Dongzhimen Hospital. The information provided by the patients will only be shared with members of the research team. Every effort will be made to keep patient information confidential. All research-related paper documents will be kept in a locked cabinet. All patient information will be kept strictly confidential.

## Trial status

Approval documents: the vision of study protocol (V1.0, 2018.12.20), informed consent (V2.0, 2019.03.26), case report form, recruitment advertisement, and copy of the main researcher GCP training certificate. At the time of submission of the manuscript, the study is in progress. We expect to complete the recruitment of subjects on December 31 of the same year.

## Supplementary information

**Additional file 1: Table S1.** SPIRIT 2013 checklist: recommended items to address in a clinical trial protocol and related documents. **Table S2.** Checklist for items in STRICTA 2010. **Table S3.** Checklist to ensure standardization of acupuncture treatment. **Figure 2.** Study design flow chart.

## Data Availability

Not applicable.

## Abbreviations

AE: Adverse reaction
CRF: Case report form
MANOVA: Multivariate analysis of variance
MOCA: Montreal Cognitive Assessment Scale
MBI: Modified Barthel Index Rating Scale
MCI: Mild cognitive impairment
MMSE: Mini Mental State Examination Scale
PSCI: Post-stroke cognitive impairment
PRECIS-2: Pragmatic Explanatory Continuum Indicator Summary Framework-2 Recommendations for Interventional Trials
SOP: Standard operation procedures
SS-QOL: Stroke-specific quality of life scale
SPIRIT: Standard Protocol Items: Recommendations for Interventional Trials
TCM: Traditional Chinese medicine
